# Supplementary material for: Endothelial Notch signaling controls insulin transport in muscle
Source: EMBO Mol Med. 2020 Mar 18;12(4):e09271. doi: 10.15252/emmm.201809271 (PMC7136962; doi:10.15252/emmm.201809271)
Supplement: Supplementary file 1 — Expanded View Figures PDF [file EMMM-12-e09271-s001.pdf]

Expanded View Figures

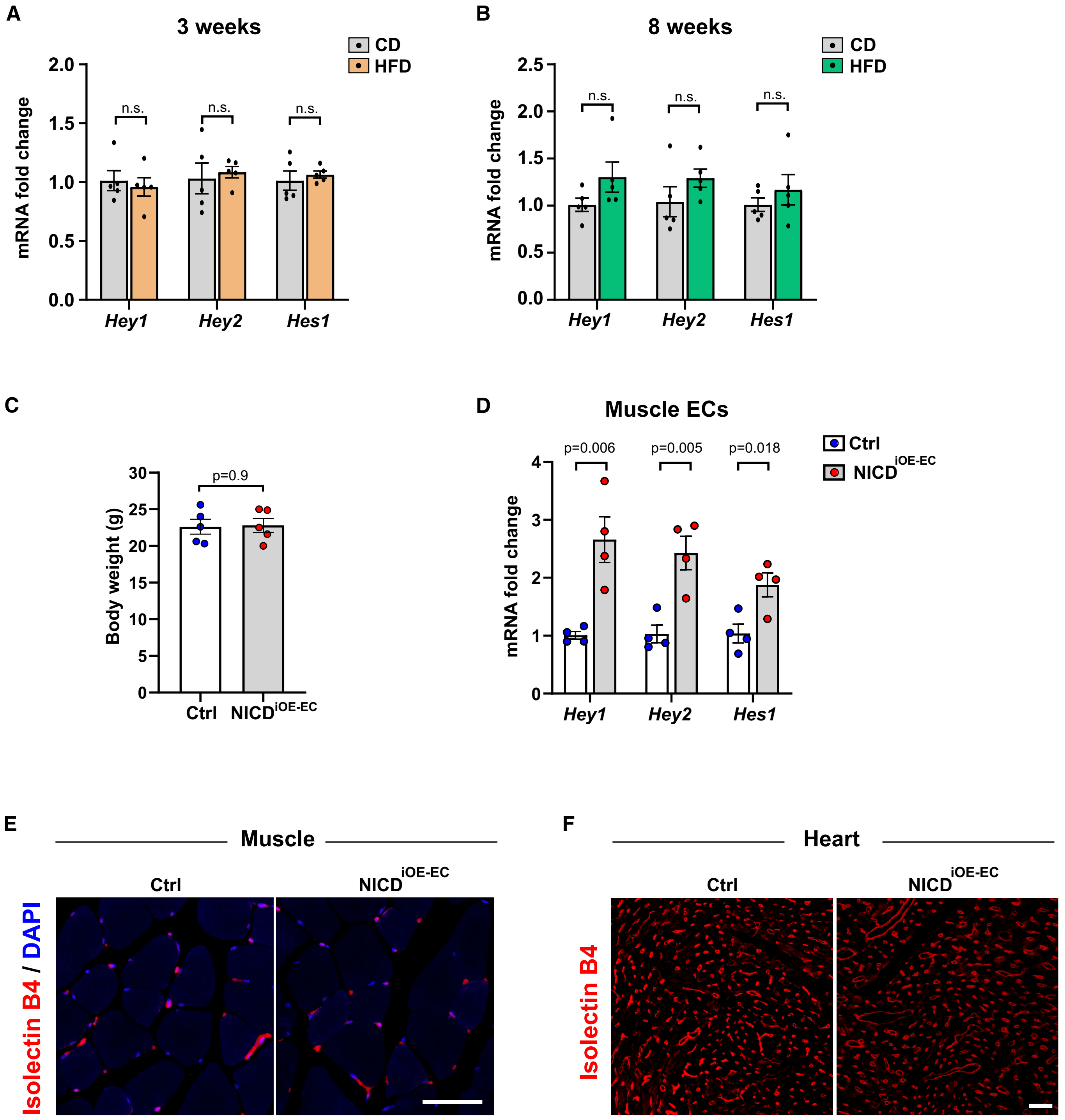

Figure EV1.

 © 2020 The Authors

◀ **Figure EV1. Notch targets in high-fat diet fed mice and characterization of NICD$^{iOE\text{-}EC}$ mice.**

A, B Expression of endothelial Notch target genes in microvascular endothelial cells isolated from skeletal muscle of mice kept on control diet (CD, 10% fat, 70% carbohydrates) or high-fat diet (HFD, 60% fat, 20% carbohydrates) for 3 weeks (A) and 8 weeks (B). n = 5, data represent mean ± SEM, unpaired t-test.

C Body mass of control (n = 5) or NICD$^{iOE\text{-}EC}$ (n = 5) mice 7 weeks after tamoxifen injection. Data represent mean ± SEM, unpaired t-test.

D Expression of Notch target genes in primary microvascular ECs freshly isolated from skeletal muscle of NICD$^{iOE\text{-}EC}$ mice compared to control mice. n = 4, data represent mean ± SEM, unpaired t-test.

E Representative confocal images of isolectin B4-stained blood vessels (red) in skeletal muscle of NICD$^{iOE\text{-}EC}$ mice and littermate controls 5 weeks after tamoxifen injection. Scale bar 50 µm.

F Representative confocal images of isolectin B4-stained blood vessels (red) in cardiac muscle of NICD$^{iOE\text{-}EC}$ mice and littermate controls 5 weeks after tamoxifen injection. Scale bar 30 µm.

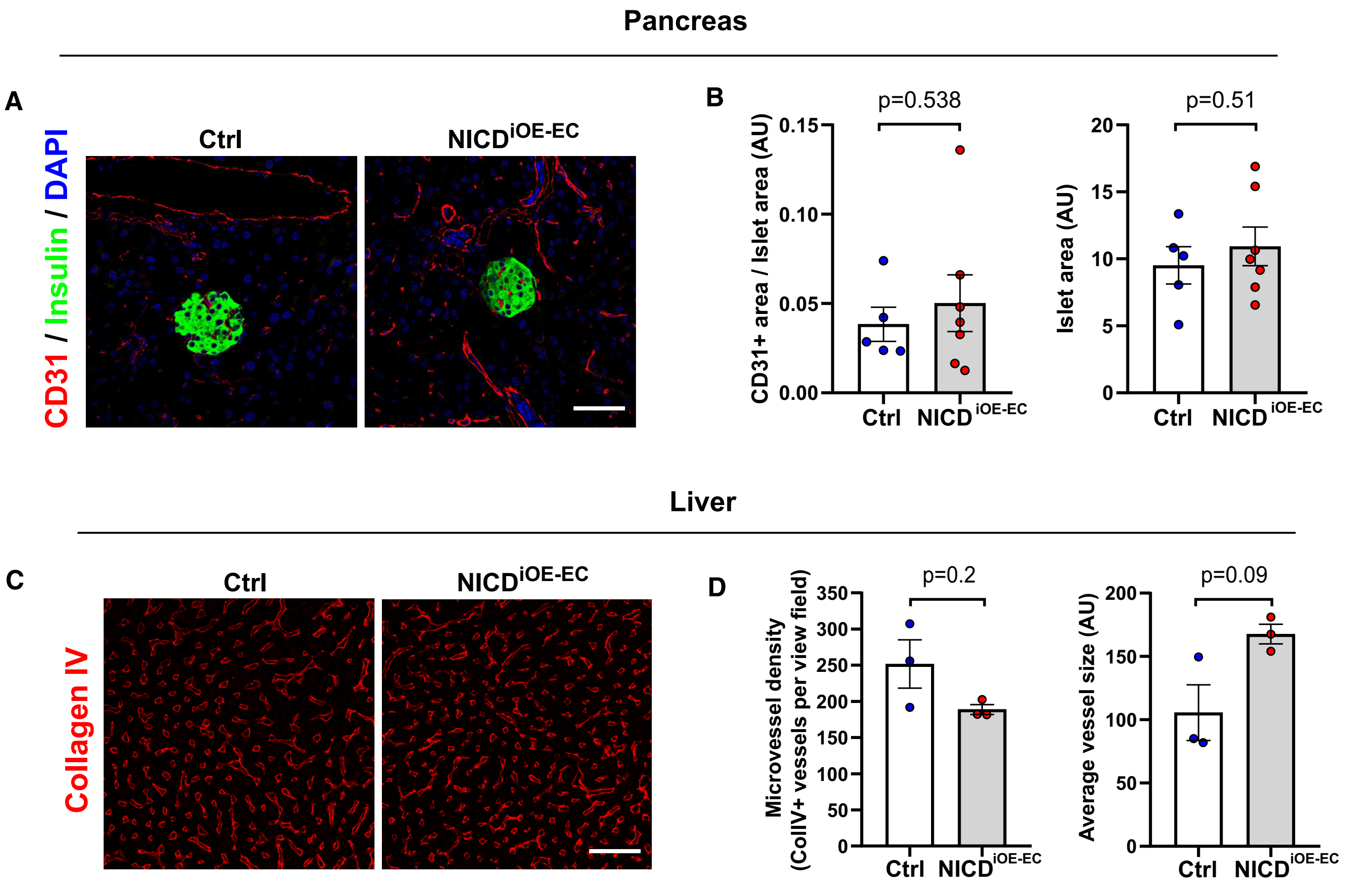

Figure EV2. Pancreas and liver vasculature in NICD$^{iOE\text{-}EC}$ mice.

A Representative confocal images showing CD31^{+} blood vessels (red) and insulin-positive (green) islets in pancreas sections from NICD$^{iOE\text{-}EC}$ mice and littermate controls 5 weeks after tamoxifen injection. Scale bar 50 µm.

B Quantification of blood vessel and islet area in control (n = 5) and NICD$^{iOE\text{-}EC}$ (n = 7) mice. Data represent mean ± SEM, unpaired t-test.

C Representative confocal images showing collagen IV+ blood vessels (red) in liver sections from NICD$^{iOE\text{-}EC}$ mice and littermate controls 5 weeks after tamoxifen injection. Scale bar 50 µm.

D Quantification of microvessel density and average vessel size in control (n = 3) and NICD$^{iOE\text{-}EC}$ (n = 3) mice. Data represent mean ± SEM, Mann–Whitney, and Welch's t-test.

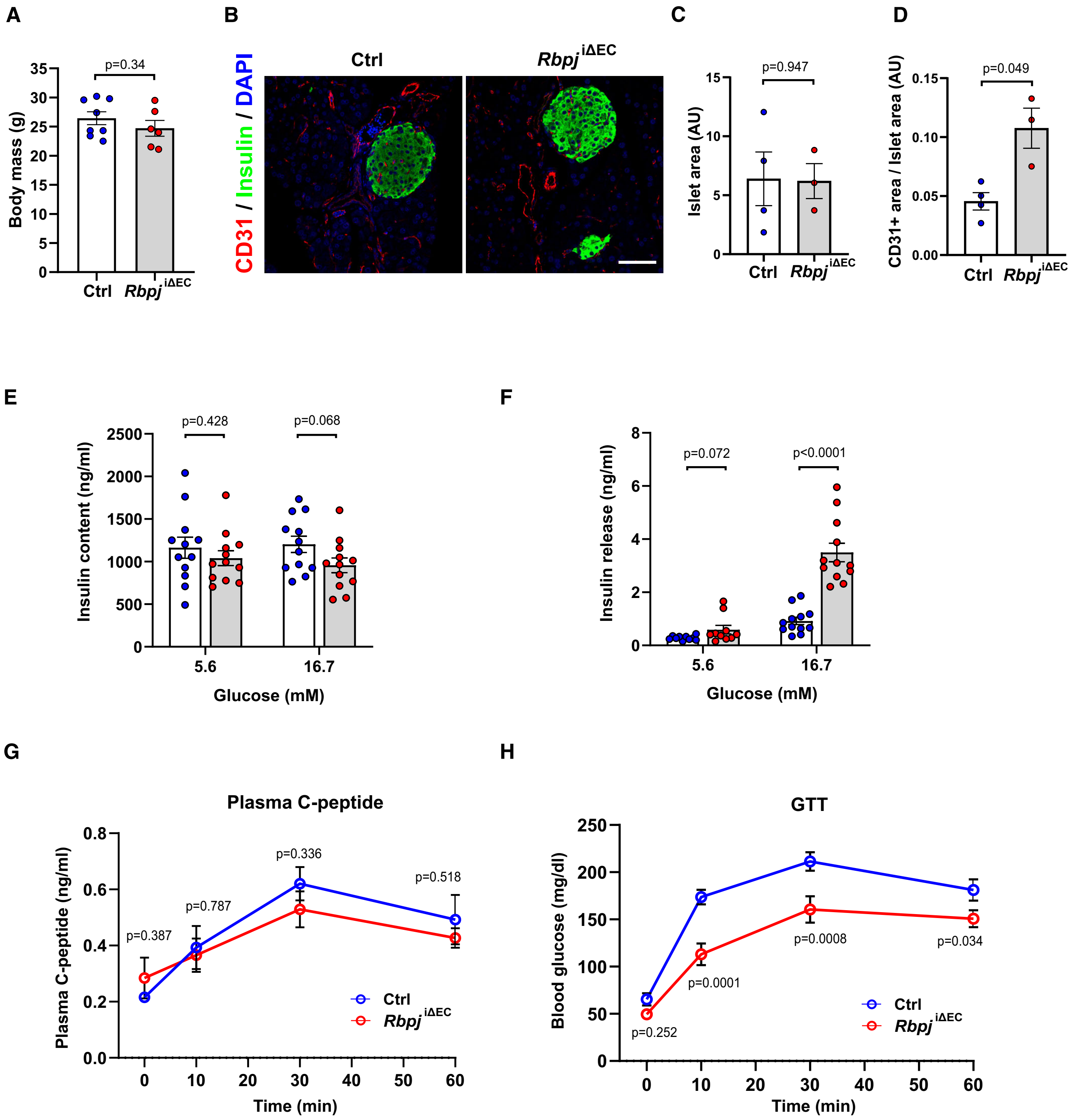

Figure EV3. Characterization of vasculature and pancreas function in *Rbpj*$^{i\Delta EC}$ mice.

A Body mass of control (n = 8) or *Rbpj*$^{i\Delta EC}$ (n = 6) mice 7 weeks after tamoxifen injection. Data represent mean ± SEM, unpaired *t*-test.

B Representative confocal images showing CD31^{+} blood vessels (red) and insulin-positive (green) islets in pancreas sections from *Rbpj*$^{i\Delta EC}$ mice and littermate controls 6 weeks after tamoxifen injection. Scale bar 50 µm.

C, D Quantification of islet area (C) and blood vessel area (D) in control (n = 4) and *Rbpj*$^{i\Delta EC}$ (n = 3) mice. Data represent mean ± SEM, Welch's *t*-test.

E, F Total insulin content (E) and *ex vivo* glucose stimulated insulin secretion (F) from pancreatic islets isolated from control (n = 5) and *Rbpj*$^{i\Delta EC}$ (n = 4) mice. Data represent mean ± SEM, unpaired *t*-test.

G, H Plasma C-peptide levels (G) and blood glucose levels (H) in control and *Rbpj*$^{i\Delta EC}$ mice after glucose stimulation. n = 4, data represent mean ± SEM, unpaired *t*-test.

© 2020 The Authors

Figure EV4. Analysis of liver vasculature and function in *Rbpj*$^{i\Delta EC}$ mice.

A Representative confocal images showing collagen IV+ blood vessels (red) in liver sections from *Rbpj*$^{i\Delta EC}$ mice and littermate controls 6 weeks after tamoxifen injection. Scale bar 50 μm.

B Quantification of microvessel density and average vessel size in control (n = 3) and *Rbpj*$^{i\Delta EC}$ (n = 3) mice. Data represent mean ± SEM, Welch's t-test.

C–E Representative images of hematoxylin and eosin (H&E) (C), Sirius red (D), and Prussian blue (E) staining of liver sections from control and NICD$^{iOE\text{-}EC}$ mice. n = 4, scale bar 100 μm.

F, G Plasma levels of albumin (F) and urea (G) in control and *Rbpj*$^{i\Delta EC}$ mice. n = 4, data represent mean ± SEM, unpaired t-test.

H–J Plasma levels of alanine aminotransferase (ALAT) (H), aspartate aminotransferase (ASAT) (I), and alkaline phosphatase (ALP) (J) in control and *Rbpj*$^{i\Delta EC}$ mice. n = 4, data represent mean ± SEM, unpaired t-test.

© 2020 The Authors

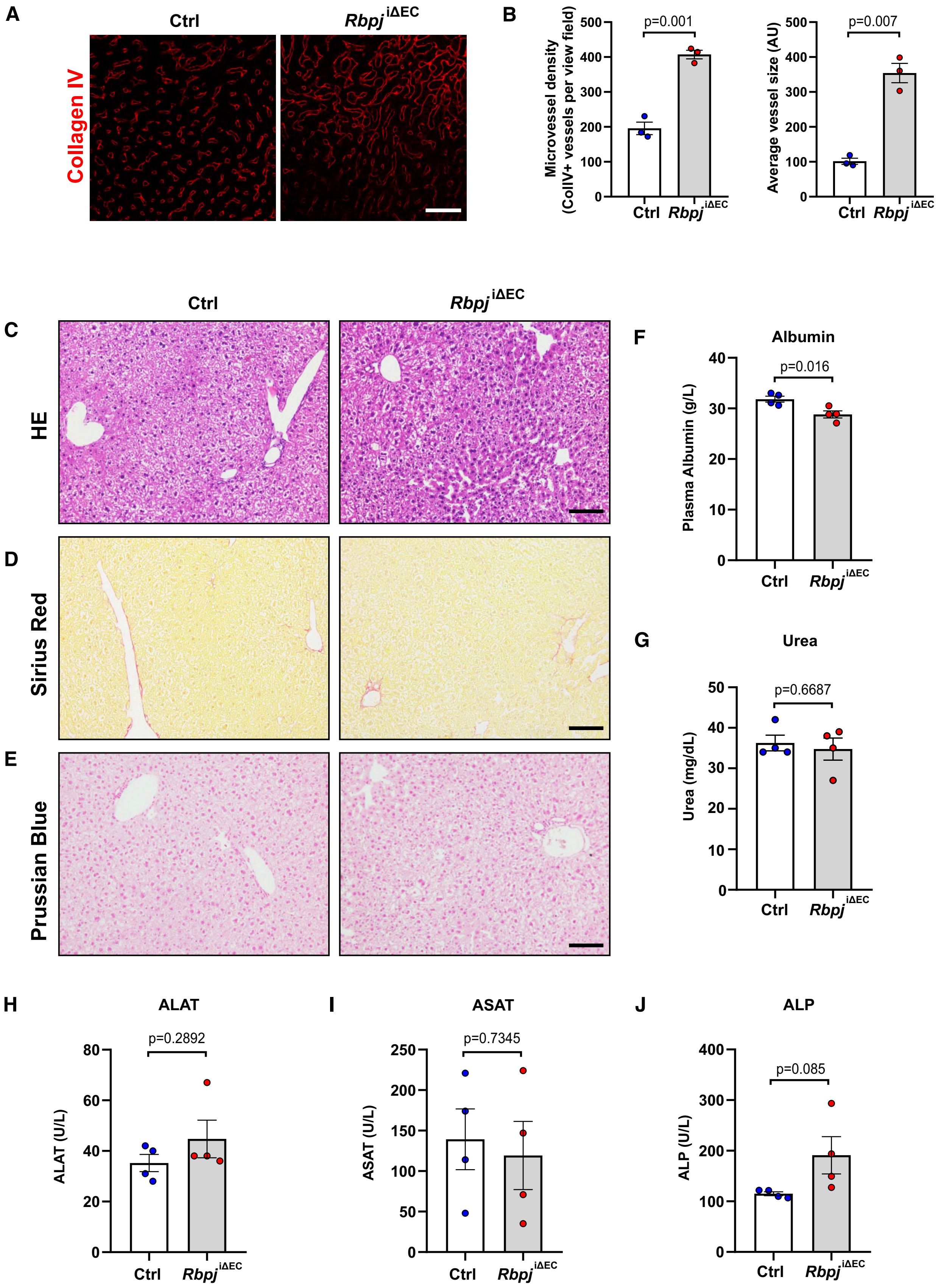

Figure EV4.

© 2020 The Authors

Figure EV5. Analysis of caveolar genes in HUVECs and *Rbpj*$^{i\Delta EC}$ mice on different diets.

- A Quantitative RT–PCR detection of *CAVIN2* and *CAVIN3* in primary human umbilical venous endothelial cells (HUVECs) upon Notch blockade (dnMAML) and induction (NICD). n = 5, data represent mean ± SEM, unpaired t-test.
- B Expression of *HEY1, HEY2, and HES1* in HUVECs upon Notch manipulation. n = 4, data represent mean ± SEM, unpaired t-test.
- C Representative Western blot of CAV2 expression in HUVECs upon Notch manipulation.
- D Densitometric analysis of Western blot in (C). Data represented as fold change over LacZ control. n = 5, data represent mean ± SEM, unpaired t-test.
- E Schematic illustration of feeding and recombination protocol.
- F Weight curve of control or *Rbpj*$^{i\Delta EC}$ mice kept on control diet (CD, 10% fat, 70% carbohydrates) or high-fat diet (HFD, 60% fat, 20% carbohydrates). n = 4 control CD, n = 5 *Rbpj*$^{i\Delta EC}$ CD, n = 6 control HFD, n = 4 *Rbpj*$^{i\Delta EC}$ HFD, data represent mean ± SEM.
- G Blood glucose levels of control (n = 9) or *Rbpj*$^{i\Delta EC}$ (n = 9) mice kept on CD. Data represent mean ± SEM, unpaired t-test.
- H Blood glucose levels of control (n = 11) or *Rbpj*$^{i\Delta EC}$ (n = 10) mice kept on HFD. Data represent mean ± SEM, unpaired t-test.

© 2020 The Authors

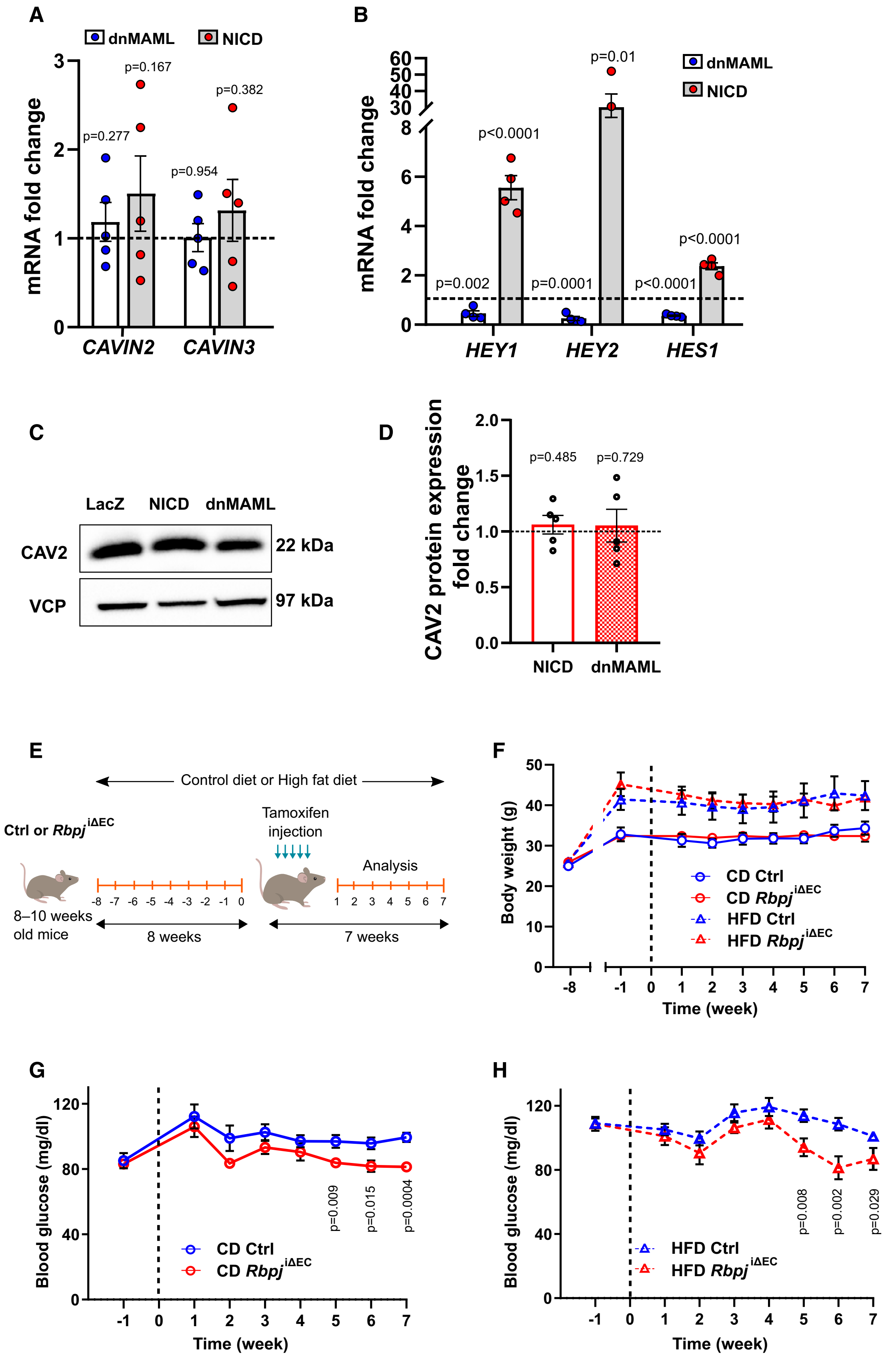

Figure EV5.

© 2020 The Authors
