# Supplementary figures and images for: Endothelial Notch signaling controls insulin transport in muscle
Source: EMBO Mol Med. 2020 Mar 18;12(4):e09271. doi: 10.15252/emmm.201809271 (PMC7136962; doi:10.15252/emmm.201809271)

Muscle

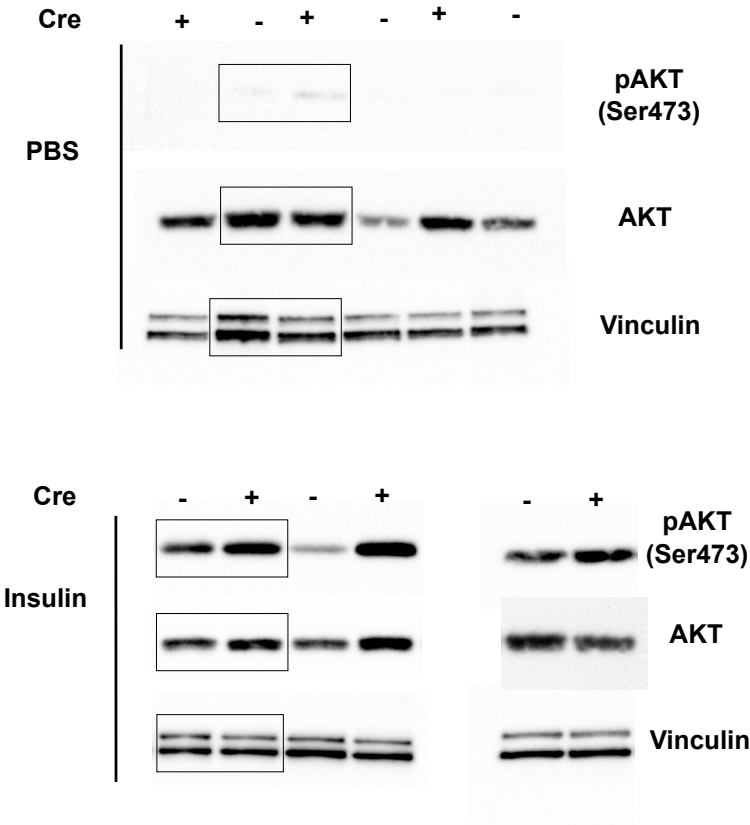

Liver

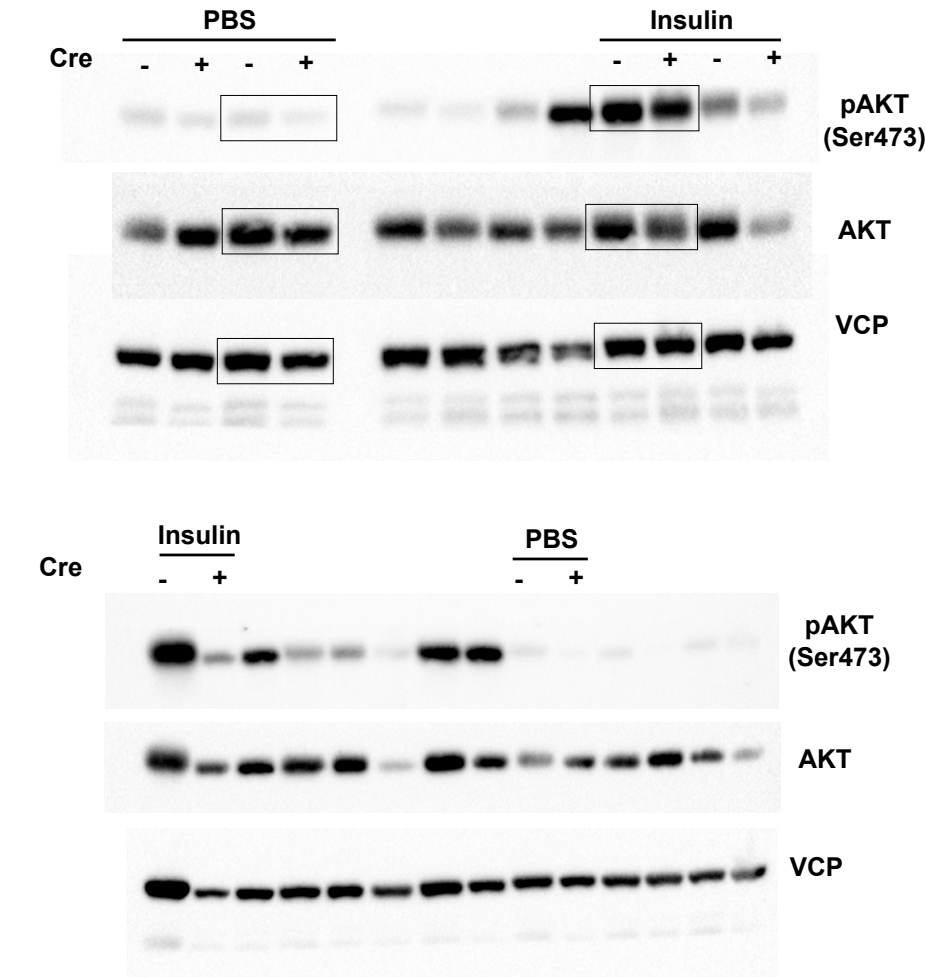

Supplement: Supplementary file 3 — Source Data for Figure 3 [file EMMM-12-e09271-s002.pdf]

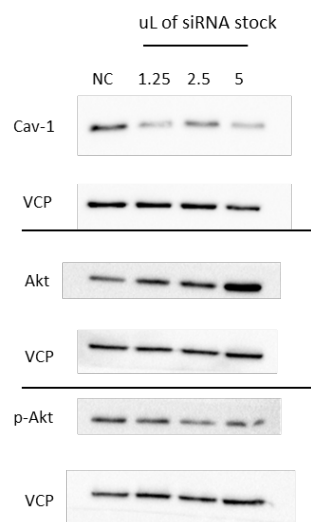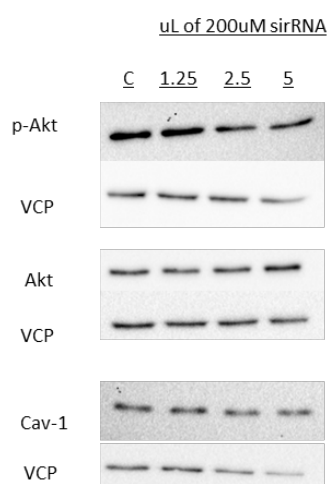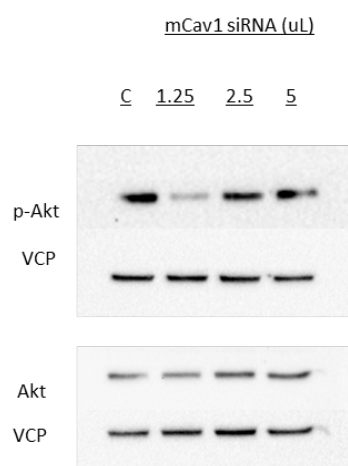

Supplement: Supplementary file 4 — Source Data for Figure 4 [file EMMM-12-e09271-s003.pdf]

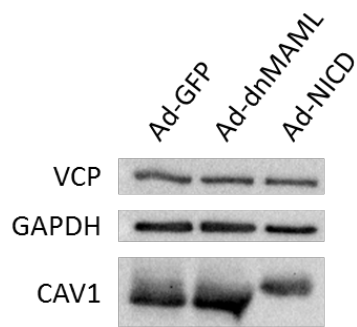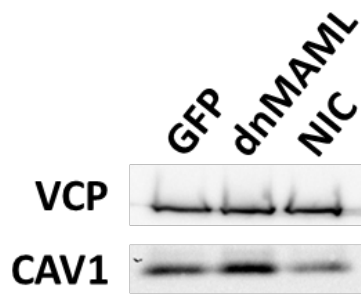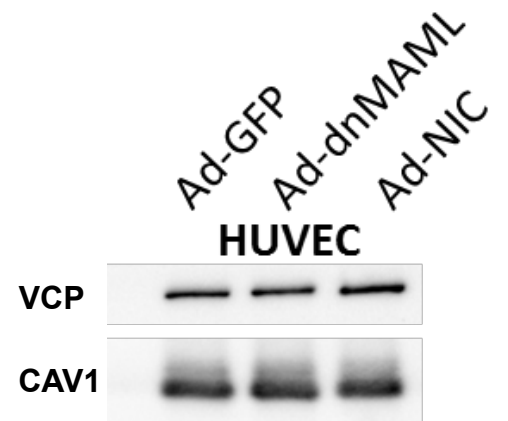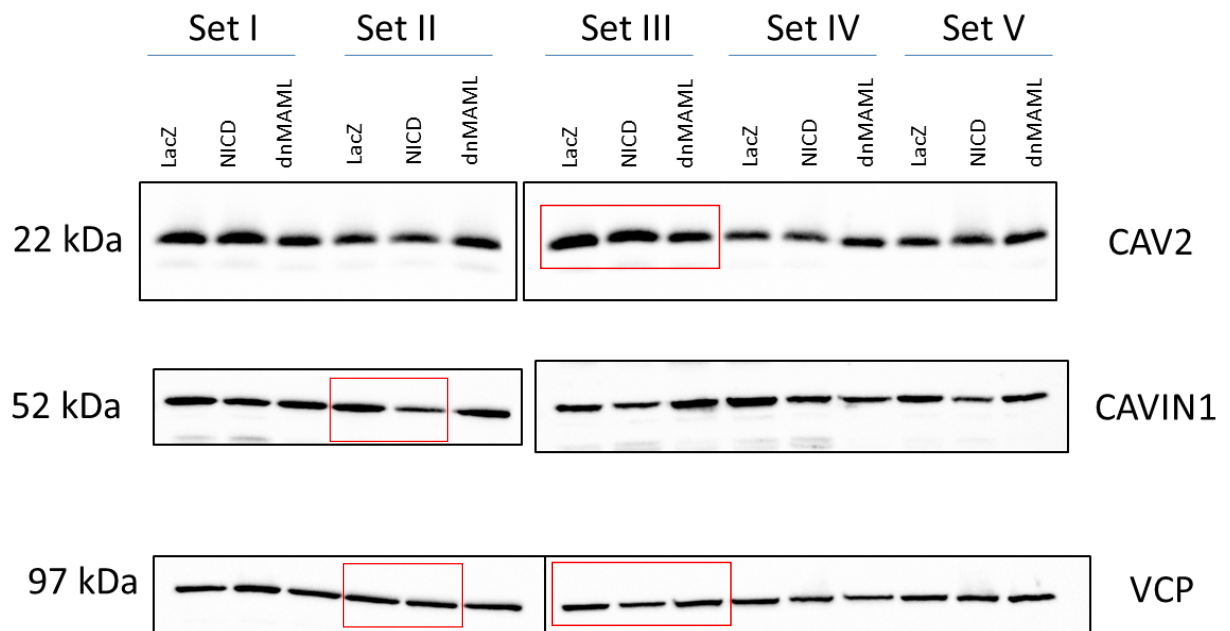

Supplement: Supplementary file 5 — Source Data for Figure 5 [file EMMM-12-e09271-s004.pdf]
